# Investigations of brain-wide functional and structural networks of dopaminergic and CamKIIα-positive neurons in VTA with DREADD-fMRI and neurotropic virus tracing technologies

**DOI:** 10.1186/s12967-023-04362-6

**Published:** 2023-08-14

**Authors:** Ning Zheng, Zhu Gui, Xiaodong Liu, Yang Wu, Huadong Wang, Aoling Cai, Jinfeng Wu, Xihai Li, Challika Kaewborisuth, Zhijian Zhang, Qitian Wang, Anne Manyande, Fuqiang Xu, Jie Wang

**Affiliations:** 1 Key Laboratory of Magnetic Resonance in Biological Systems, State Key Laboratory of Magnetic Resonance and Atomic and Molecular Physics, National Center for Magnetic Resonance in Wuhan, Wuhan Institute of Physics and Mathematics, Innovation Academy for Precision Measurement Science and Technology, Chinese Academy of Sciences-Wuhan National Laboratory for Optoelectronics, 430071 Wuhan, People’s Republic of China; 2 University of Chinese Academy of Sciences, Beijing, 100049 China; 3 Department of Anaesthesia and Intensive Care, Peter Hung Pain Research Institute, The Chinese University of Hong Kong, Hong Kong, SAR China; 4 Shenzhen Key Laboratory of Viral Vectors for Biomedicine, Key Laboratory of Quality Control Technology for Virus-Based Therapeutics, Guangdong Provincial Medical Products Administration, NMPA Key Laboratory for Research and Evaluation of Viral Vector Technology in Cell and Gene Therapy Medicinal Products, The Brain Cognition and Brain Disease Institute (BCBDI), Shenzhen Institute of Advanced Technology, Chinese Academy of Sciences, Shenzhen-Hong Kong Institute of Brain Science-Shenzhen Fundamental Research Institutions, Shenzhen, 518055 China; 5 College of Integrative Medicine, Fujian University of Traditional Chinese Medicine, Fuzhou, Fujian People’s Republic of China; 6 Virology and Cell Technology Research Team, National Center for Genetic Engineering and Biotechnology (BIOTEC), National Science and Technology Development Agency (NSTDA), Pathumthani, 12120 Thailand; 7 Department of Neuroscience and Mahoney Institute for Neurosciences, Perelman School for Medicine, University of Pennsylvania, Philadelphia, PA 19104 USA; 8 School of Human and Social Sciences, University of West London, Middlesex, TW8 9GA UK; 9 Institute of Neuroscience and Brain Diseases, Xiangyang Central Hospital, Affiliated Hospital of Hubei University of Arts and Science, Xiangyang, Hubei People’s Republic of China

**Keywords:** Ventral tegmental area (VTA), Neural networks, DREADD, fMRI, HSV

## Abstract

**Background:**

The ventral tegmental area (VTA) contains heterogeneous cell populations. The dopaminergic neurons in VTA play a central role in reward and cognition, while CamKIIα-positive neurons, composed mainly of glutamatergic and some dopaminergic neurons, participate in the reward learning and locomotor activity behaviors. The differences in brain-wide functional and structural networks between these two neuronal subtypes were comparatively elucidated.

**Methods:**

In this study, we applied a method combining Designer Receptors Exclusively Activated by Designer Drugs (DREADD) and fMRI to assess the cell type-specific modulation of whole-brain neural networks. rAAV encoding the cre-dependent hM3D was injected into the right VTA of DAT-cre or CamKIIα-cre transgenic rats. The global brain activities elicited by DREADD stimulation were then detected using BOLD-fMRI. Furthermore, the cre-dependent antegrade transsynaptic viral tracer H129ΔTK-TT was applied to label the outputs of VTA neurons.

**Results:**

We found that DREADD stimulation of dopaminergic neurons induced significant BOLD signal changes in the VTA and several VTA-related regions including mPFC, Cg and Septum. More regions responded to selective activation of VTA CamKIIα-positive neurons, resulting in increased BOLD signals in VTA, Insula, mPFC, MC_R (Right), Cg, Septum, Hipp, TH_R, PtA_R, and ViC_R. Along with DREADD-BOLD analysis, further neuronal tracing identified multiple cortical (MC, mPFC) and subcortical (Hipp, TH) brain regions that are structurally and functionally connected by VTA dopaminergic and CamKIIα-positive neurons.

**Conclusions:**

Our study dissects brain-wide structural and functional networks of two neuronal subtypes in VTA and advances our understanding of VTA functions.

**Supplementary Information:**

The online version contains supplementary material available at 10.1186/s12967-023-04362-6.

## Background

The ventral tegmental area (VTA) and its connections are the critical parts of the reward system and play important roles in various functions, such as learning, motivation and addiction [1, 2]. These behaviors are mainly mediated by dopamine release in the downstream regions of VTA dopaminergic neurons [1]. The projections from VTA dopaminergic neurons target many anterior cortical regions, including the prefrontal cortex (PFC), nucleus accumbens (NAcc), dorsal striatum and amygdala (AMY) [3]. The VTA dopamine neurons projected to NAcc and PFC are referred to mesolimbic and mesocortical pathways, respectively. The mesolimbic pathway is mainly involved in the regulation of motivation, reward, reinforcement learning and other cognitive processes. Dysfunction of mesolimbic pathway is associated with neurological diseases such as addiction, depression and schizophrenia [4]. The mesocortical pathway is related to cognitive control, motivation and emotional response. Its abnormality may cause some mental illnesses such as schizophrenia [5]. Even though more than 60% neurons in the VTA are dopaminergic, the VTA is heterogenous and contains different types of neurons other than the dopaminergic neurons, including GABAergic and glutamatergic neurons [1, 6]. Moreover, the distinction between them is not very precise. Some neurons are known to release both glutamate and dopamine [7], GABA and dopamine [7–9] or even glutamate and GABA [10, 11].

Ca^2+^/calmodulin-dependent protein kinase II alpha (CamKIIα) is a protein kinase in the second messenger pathway of the N-methyl-D-aspartic acid (NMDA) receptor. The activation of CamKIIα is regulated by calcium ions, leading to synaptic insertion, enhanced single-channel conductance of the a-amino-3-hydroxy-5-methyl-4-isoxazole propionic acid (AMPA) receptor [12–14], and other long-lasting neural changes [15]. CamKIIα, therefore, is one of the key enzymes involved in learning, memory, and synaptic plasticity [16]. Although CamKIIα is known to be expressed mainly in glutamatergic neurons, this protein also presents in some populations of dopaminergic neurons that co-release glutamate [17–19]. CamKIIα-positive neurons in the VTA contain primarily glutamatergic neurons and some dopaminergic neurons. They were demonstrated to participate in reward learning mediated behavior and induce similar rewarding effects with VTA dopaminergic neurons as demonstrated by optogenetic intracranial self-stimulation [19, 20]. However, the functional and structural networks of VTA dopamine neurons and CamKIIα-positive neurons have not been comparatively evaluated so far in the whole-brain level.

In the last decade, a development of neuromodulatory tools, especially in optogenetics and chemogenetics progressively allow the regulation of the activity of specifically genetical-targeted neuron populations with spatiotemporal specificity. The combination of optogenetics/chemogenetics and non-invasive neuroimaging methods such as functional MRI (fMRI) enables direct assessment of large-scale neural activity caused by the modulation of specifically targeted neurons, revealing the role of the targeted neurons in the brain-wide neural network. The optogenetics fMRI (ofMRI) has been demonstrated to be a valuable tool that can map the VTA-activation related functional network. According to recent ofMRI studies, stimulation of dopaminergic VTA neurons leads to increase in blood oxygenation level dependent (BOLD) signal intensities mostly in the dorsal and ventral striatum, pallidum and thalamus (TH) [21–24]. However, some studies have indicated that only weak and sparse BOLD responses are elicited by the optogenetic stimulation of VTA dopaminergic neurons, and that significant functional responses are absent in the well-known reward-related areas such as the dorsal and ventral striatum [20, 25]. Their results also showed that the excitation of VTA CamKIIα-positive neurons caused BOLD signal changes in multiple regions, such as the ventral striatum, PFC, and TH. Thus, the results of the ofMRI studies of VTA dopaminergic remain controversial.

The combination of Designer Receptors Exclusively Activated by Designer Drugs (DREADD) and fMRI, so called DREADD-fMRI, could also be utilized to evaluate large-scale network activity in response to the excitation of targeted neurons in a specific region [26–29]. For DREADDs, the engineered receptors allow targeted activation or silencing of neurons upon binding to the specific ligand [30]. DREADD-fMRI has been utilized to map the functional networks related to the VTA. It was found that the DREADD activation of VTA neurons involved in mesolimbic or mesocortical pathways, respectively, induced significant BOLD responses in DREADD-expressing regions and in the neural circuit without DREADD expression, such as Amygdala, NAcc, mPFC, ventral Pallidum (VP), TH and Insula [29]. However, the DREADD stimulation in this study was not cell type-specific. To the best of our knowledge, there is no DREADD-fMRI study of specific cell-type neurons in the VTA.

In this study, we aim to investigate the functional and structural neural networks of the specific type neurons in the VTA. DREADD-fMRI was employed to map the whole-brain neural network activity upon the activation of dopaminergic or CamKIIα-positive neurons in the VTA. To comprehensively compare the functional network related to these two different types of neurons in the VTA, the brain-wide fMRI activation maps and BOLD signal time courses after VTA dopaminergic or CamKIIα-positive neurons stimulation were analyzed. Moreover, herpes simplex virus (HSV), a trans-multi-synaptic virus was applied to trace the downstream structural networks of VTA dopaminergic neurons and CamKIIα-positive neurons. Comparative study of structural and functional neural networks of these different cell-type subpopulations contributes to a deeper understanding of the brain-wide neural networks of VTA.

## Methods

### Animals

The transgenic rats, DAT-cre and CamKIIα-cre strains (Beijing Biositu Gene Biotechnology, Beijing, China), were mated with Sprague Dawley (SD) rats (Hunan SJA Laboratory Animal, China). The dopaminergic or CamKIIα-positive neurons of DAT-cre/CamKIIα-cre transgenic rats expressed cre recombinase, and male rats (8 to 12-week-old) were used in the current experiments. All animals were housed under the standard humidity and temperature conditions with a light/dark cycle of 12/12 h, and food and water were provided ad libitum.

### Stereotaxic surgery

Adeno-associated virus vector (AAV), rAAV-hSyn-DIO-hM3D(Gq)-mCherry-WPRE-pA (rAAV-DIO-hM3D(Gq)-mCherry, 6.9E+12 vg/ml, Brain Case, Shenzhen, Guangdong, China) was used to mediate the chemogenetics activation, and the trans-multi-synaptic HSV vector, H129ΔTK-TT (6.4E+09 PFU/ml, Brain Case) was used for tracing the structural networks [31].

On the experimental day, the DAT-cre (N = 5) or CamKIIα-cre (N = 4) rats were anesthetized with sodium pentobarbital (40 mg/kg, *i.p.*) and mounted onto a stereotaxic holder (68030, RWD, Shenzhen, Guangdong, China) for the stereotaxic injection into the target brain region [ventral tegmental area (VTA): A-P, − 5.0 mm; M-L, − 0.8 mm, D-V, − 8.1 mm]. For the chemogenetics experiments, the virus of rAAV-DIO-hM3D(Gq)-mCherry (300 nl) was injected into the right VTA of brain. The rats were submitted to behavioral experiments (a detailed description can be found in the Additional file 1) and fMRI scanning after four weeks’ virus expression, then the animal was perfused, and the brain was obtained for sectioning and immunohistochemical staining to verify the virus expression. For the structural neural circuit tracing, the virus of H129ΔTK-TT (200 nl) was injected into the right VTA. The rat was perfused 3 days later and then the brain was obtained for sectioning and immunohistochemical staining to investigate the structural networks.

### MRI experiments

The MRI experiments were all completed in a Bruker's horizontal 7.0T small animal scanner (Bruker, Germany). The transmitting coil was a birdcage body coil, and the receiving coil was a 20-mm-diameter open ring surface coil. The animals were initially anesthetized with 3.0–5.0% isoflurane, and then they were injected with 7.5 μg/ml dexmedetomidine hydrochloride solution (0.83 ml/kg, *i.p.*). The sedative, dexmedetomidine hydrochloride, helps to maintain the animals in a steady state during the MRI experiments. Then, the rat was fixed on the MRI animal bed, and the injection needle was connected to the catheter and embedded in the abdomen of the fixed animal, which was used for Clozapine N-oxide (CNO) or saline infusion (the same dosage as the animal behavioral experiment) during the MRI scanning. A needle connected to the catheter was buried subcutaneously at the back of the rat, and was used to infuse dexmedetomidine hydrochloride (8.3 μl/min/kg) through a micro-injection pump during the MRI experiment. Furthermore, a circulating hot water bath was used to maintain the animal body temperature, and the rectal temperature was monitored by a digital thermometer with a probe. The breathing rate of the animals was monitored by a respiration monitoring system.

The scanning range and resolution used for the MRI acquisition are as follows: field of view (FOV) = 34 mm × 34 mm; matrix size = 96 × 96; spatial resolution = 0.354 mm × 0.354 mm; slice thickness = 0.8 mm. The sequence used for the BOLD fMRI is a multi-layer FLASH sequence, and the parameters are as follows: TR = 500 ms; TE = 13.5 ms; FA = 30°; NA = 2; number of repetitions = 65; total scanning time = 104 min. The sequence used in the T2-weighted structural image is RARE sequence, and the parameters are as follows: TR = 3000 ms; TEeff = 36 ms; NA = 4; total scan time = 12 min 48 s.

### Slice preparation and immunohistochemistry

Rats were anesthetized with an overdose of sodium pentobarbital (50 mg/kg, *i.p.*) and then transcardially perfused with phosphate buffered saline (PBS) and 4% paraformaldehyde (Sigma, USA). Then, the brain was removed and fixed in 4% PFA overnight at 4 °C. Following dehydration with 30% sucrose, 40 μm coronal brain sections were prepared with a microtome (Thermo Fisher Scientific, Waltham, MA, USA). The brain sections were washed with PBS (three times and 5 min for each wash), then the slices were incubated with the blocking solution (10% normal goat serum and 0.3% Triton x-100 in PBS) for 1 h at 37 °C, followed by rabbit primary antibody at 4 °C for 24 h. Sections were washed three times with PBS (5 min per each wash) and incubated with secondary goat anti-rabbit cy3 or 488 for 1 h at 37 °C, stained with 4′,6-diamidino-2-phenylindole (DAPI), washed with PBS three times (5 min per wash), and mounted with 70% glycerol. The brain slices were imaged under a confocal microscope (TCS SP8, Leica, Deerfield, IL, USA) or a virtual microscopy slide-scanning system (VS 120, Olympus, Tokyo, Japan). The fluorescence images of different color channels are imported into ImageJ for overlay and the addition of a scale bar. The antibody information used in this experiment was collected in the supplementary materials (Additional file 1: Table S1).

### Data analysis

The raw MRI data was converted to nifti format by Bruker2Analyze Converter. The brains in the T2-weighted structural images were manually extracted using MRIcron software (www.nitrc.org/projects/mricron/). The brain masks of the structural images were utilized to extract the brains of BOLD fMRI images. The motion correction of BOLD fMRI images was performed using SPM12 (www.fil.ion.ucl.ac.uk/spm). Realignment was performed through a 6-parameter rigid body spatial transformation. Afterword, both the structural images and BOLD fMRI images were normalized to a homemade rat brain anatomical MRI template using SPM12 using a 12-parameter affine transformation, followed by a non-linear transformation. Then, the spatial smoothing was conducted with a Gaussian kernel with full width at half maximum of twice the voxel size. The region of the VTA was segmented based on SIGMA rat brain atlas [32] and the spreading areas of mCherry fluorescent signals. The voxel-wise area under the curve (AUC) of the time course for the activation phase (volumes 45–65) was calculated and compared by the 3dttest++ function in AFNI (https://afni.nimh.nih.gov/). The *t* value map was then overlapped on the homemade template. To compare the activation maps across different cell type-specific stimulations, the conjunction analysis was performed to identify significant voxels in at least one stimulation or both stimulations. The activated areas were segmented in different brain regions ROI(s) according to the SIGMA brain atlas, and the mean time courses of the ROI(s) were extracted. The changes in BOLD signal intensity are presented as percentage change from the baseline (averaged intensity across the fifth to the twenty-fifth scan). The time courses of BOLD signals in different ROI(s) were fitted to sigmoidal curves (Eq. 1), and the increase values (parameter ‘*a’* in Eq. 1) were obtained via the fitting. By conducting two-sample *t*-tests on the increase values of different brain regions pairwise, a comparison matrix was obtained. Different colors represent the *p*-values indicating the significance of the comparison between increase values. No significant difference between increase values is displayed as dark blue. The warmer the color, the more significant the differences between the two regions.

1 $$f\left(x\right)=1+\frac{a}{1+{e}^{b(c-x)}}$$

## Results

### Expression of rAAV-DIO-hM3D(Gq)-mCherry in VTA dopaminergic neurons

**Fig. 1:**
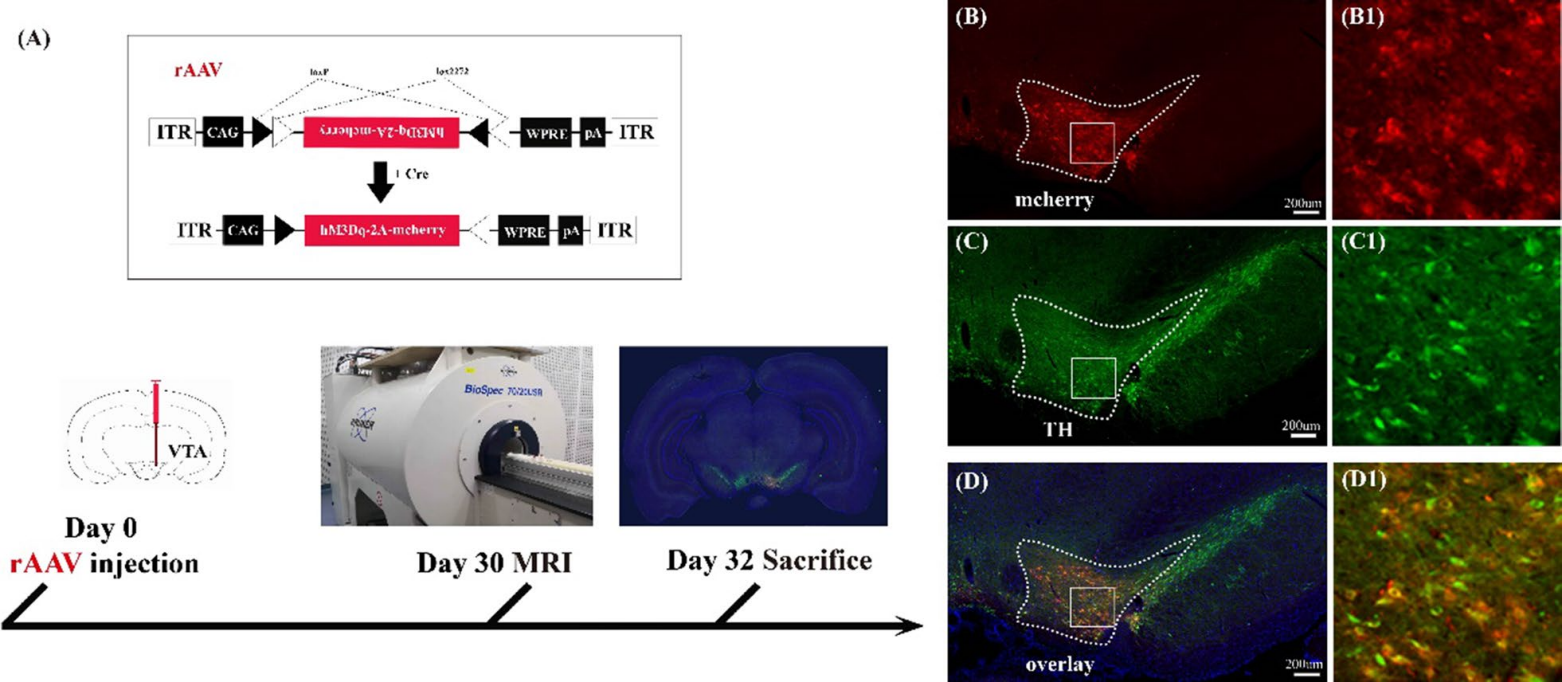
Experimental procedure for chemogenetic activation of VTA neurons and immunohistochemical fluorescence imaging. **A** rAAV encoding the cre-dependent DREADD receptor rAAV-DIO-hM3D(Gq)-mCherry was injected into the right VTA of DAT-cre transgenic rats and an experimental design shows a timeline including the virus injection, behavioral test, and MRI detection. **B–D** Confirmation of rAAV-infected dopaminergic neurons in the VTA; **B** the cre-dependent neurons expressing hM3D (Gq) and mCherry proteins (red); **C** dopaminergic neurons were stained by anti-Tyrosine Hydroxylase (green); **D** illustration of the virus-infected dopaminergic neurons (yellow) by merging the results of the virus labeling (**B**) and immunohistochemistry (**C**). Panels **B1**, **C1**, **D1** are higher-magnification images of boxed regions in **B–D**. The nuclei were stained blue by DAPI, and the scale bar: 200 μm

To specifically target and activate the VTA dopaminergic neurons, the rAAV encoding cre-dependent DREADD receptor, rAAV-DIO-hM3D(Gq)-mCherry, was injected into the right VTA region of DAT-cre transgenic rats (Fig. 1A). The hM3D(Gq) receptor can mediate the activation of neurons by the designed ligand, CNO. The expression of hM3D(Gq) receptor in neurons was indicated by the red fluorescence of mCherry protein (Fig. 1B). In order to confirm that the neurons expressing hM3D(Gq) and mCherry proteins are dopaminergic neurons, immunohistochemistry was used to visualize the localization of tyrosine hydroxylase (TH) protein in brain slices containing the virus injection site (Fig. 1C). The result indicated that TH and hM3D(Gq) was substantially colocalized, and suggested that hM3D(Gq) was specifically expressed in the dopaminergic neurons (Fig. 1D).

### Variations of c-Fos expression and animal behaviors induced by the activation of dopaminergic neurons in the VTA

**Fig. 2:**
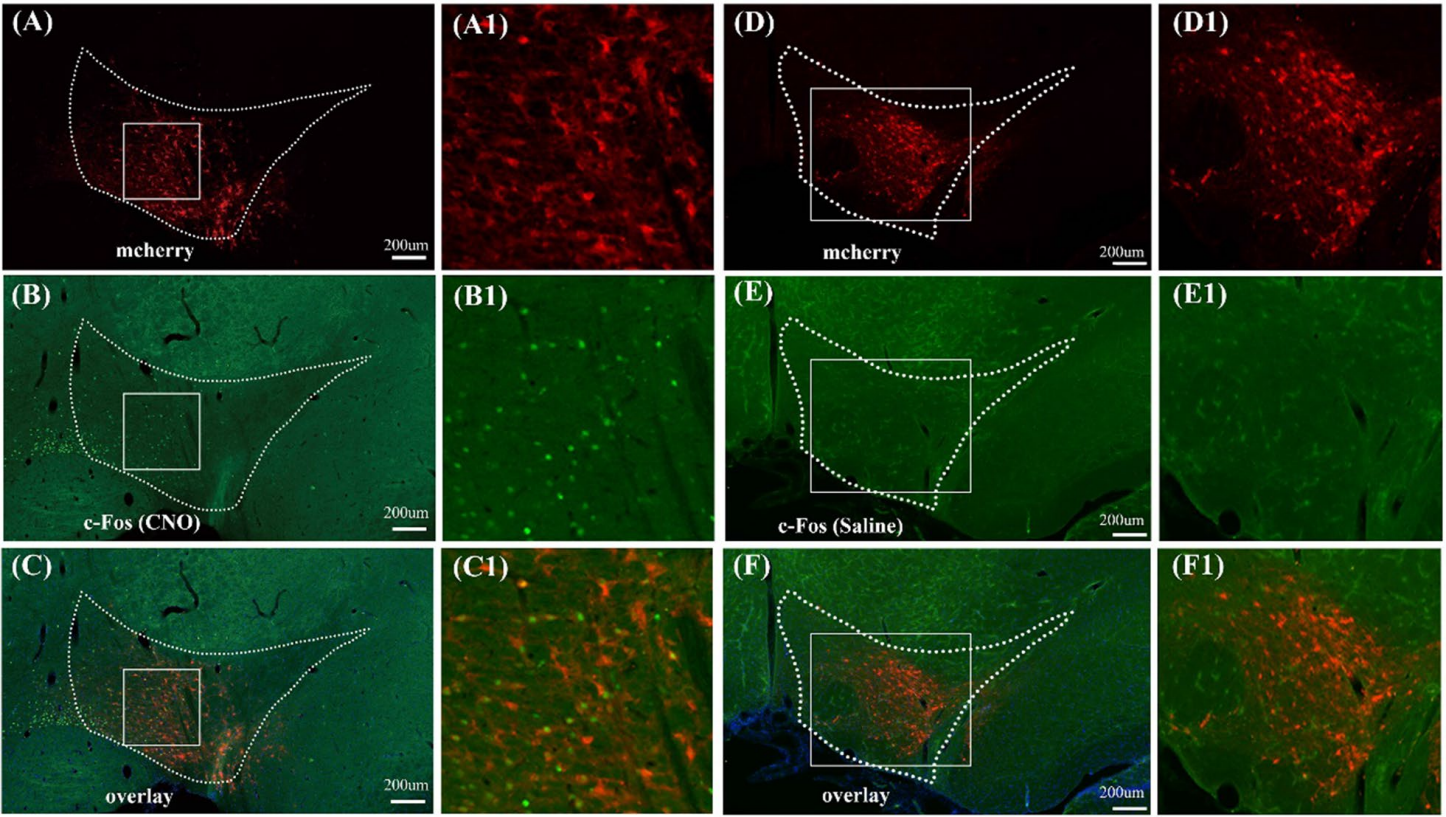
Immunofluorescent c-Fos staining in the VTA of DAT-cre rats infected with hM3D(Gq) encoding rAAV after the activation with CNO (chemogenetic activation) or saline. **A–C** Representative coronal cerebral sections show the locations of virus-labeled neurons (red, **A**), the immunofluorescent neurons with *c*-Fos staining (green, **B**) and the co-localization neurons (yellow, **C**) in the VTA after the chemogenetics activation of VTA dopaminergic neurons; Panels **A1**, **B1** and **C1** are higher-magnification images of the boxed regions in panels of **A–C**, respectively; **D–F** the locations of virus-labeled neurons (red, **D**); immunofluorescent *c*-Fos staining (green, **E**); and the merged results of the co-localization labeling (yellow, **F**). The results indicated that the saline injection did not cause obvious *c*-Fos signal in the region of VTA of DAT-cre transgenic rats; Panels **D1**, **E1** and **F1** are higher-magnification images of boxed regions in the panels of **D–F**, respectively. The nuclei were stained blue by DAPI. The scale bar: 200 μm

The c-Fos immunostaining was performed to verify the activation of VTA neurons caused by DREADD with the injection of CNO. The results indicated that the *c*-Fos expression increased in the VTA region after CNO injection in the DAT-cre rats infected with rAAV-DIO-hM3D(Gq)-mCherry (Fig. 2A–C). There was extensive overlap between the expression of c-Fos and hM3D(Gq) in VTA (Fig. 2C). No* c*-Fos expression in the VTA was detected in rats injected with saline (Fig. 2D–F). Additionally, the locomotor hyperactivity is a known behavior output of the VTA dopaminergic neurons activation [5, 33–35]. Thus, the locomotor activity of DAT-cre and CamKIIα-cre rats infected with rAAV-DIO-hM3D(Gq)-mCherry was further monitored after the CNO or saline injection. Compared with the saline injection, rats in the CNO group significantly increased in total distance travelled (Additional file 1: Figs. S3 and S4), confirming that the VTA neurons were activated through DREADD.

### Variations of the BOLD signals induced by the activation of dopaminergic neurons in the VTA

**Fig. 3:**
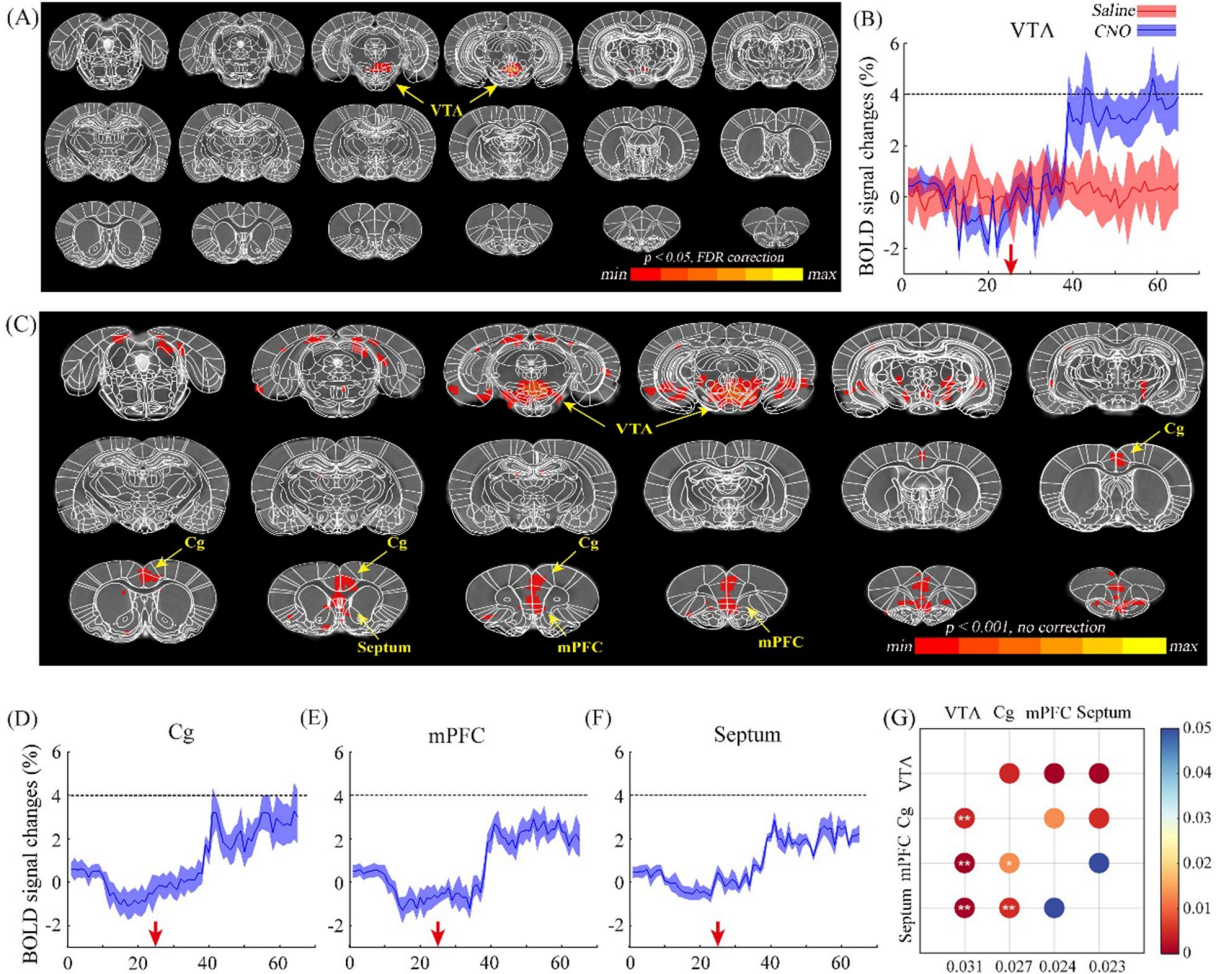
The results of BOLD response after the chemogenetic activation of VTA dopaminergic neurons in DAT-cre rats (n = 5) with CNO. **A** Global responses of BOLD signal after chemogenetic activation with FDR correction (*p*-FDR corrected < 0.05); results indicate that only the VTA shows positive signals; **B** BOLD signal changes in the virus infected region (VTA) during the chemogenetics activation of VTA dopaminergic neurons; red arrow indicates the injection timepoint of CNO (blue) or saline (red); **C** global responses of BOLD signal after chemogenetic activation without FDR correction (p < 0.001), the regions of VTA, Cg, Septum and mPFC show positive signals; **D–F** variations of BOLD signal in the regions of Cg (**D**), mPFC (**E**) and Septum (**F**) after the injection of CNO (red arrow), respectively; **G** the comparison matrix of the increased BOLD signals in the VTA, Cg, mPFC and Septum. The increase values of BOLD signals in the activated brain regions were calculated and compared pairwise. The colors of dots indicate *p*-value. Two-tailed t-test; *p < 0.05; **p < 0.01

The global brain activity through DREADD activation of VTA dopaminergic neurons was investigated using BOLD-fMRI. After 25 baseline fMRI scans, CNO was administrated through an intraperitoneal injection, followed by 40 post-injection image volumes. The whole-brain BOLD responses of the activation phase followed by CNO or saline injection were analyzed and compared voxel-by-voxel. The neural activity map induced by DREADD activation is shown in Fig. 3A and C. Unexpectedly, the BOLD response was only found in VTA region in the result corrected by FDR (Fig. 3A). DREADD activation of VTA dopaminergic neurons resulted in elevated BOLD signals in the VTA region (Fig. 3A and B), while there was no distinct BOLD response in the saline group (Fig. 3B). The BOLD signals started to increase at the 35th fMRI scan and reached the plateau around the 40th scan. Therefore, the image series from the 45th to the last were considered as the activation phase. To reduce the false negatives, the analysis was repeated without multiple comparison correction. New results demonstrated that neural activity changes were presented in several regions including the VTA, medial prefrontal cortex (mPFC), cingulate cortex (Cg) and Septum (Fig. 3C). Time courses of BOLD signals in these regions were extracted and illustrated in Fig. 3D–F. Furthermore, the increased values of BOLD signals caused by DREADD stimulation of the aforementioned four brain regions were calculated and compared. The comparison matrix indicates that the VTA exhibited the strongest BOLD response, while response amplitudes of mPFC and Septum are the smallest (Fig. 3G). This finding indicates that DREADD activation of VTA dopaminergic neurons induced neural activity in the VTA itself and the other functional related regions.

### DREADD activation of CamKIIα-positive neurons in the VTA

Similarly, VTA CamKIIα-positive neurons were targeted and investigated by the injection of rAAV-DIO-hM3D(Gq)-mCherry in the VTA region of the CamKIIα-cre transgenetic rats. The expression of hM3D(Gq) receptor in CamKIIα-positive neurons was confirmed by the combination of mCherry fluorescent imaging and CamKIIα immunostaining (Additional file 1: Fig. S1). The immunohistochemistry of c-Fos protein was also performed to verify the DREADD activation of VTA neurons (Additional file 1: Fig. S2). The results indicate that c-Fos protein expression was increased in the VTA after the CNO activation. These results demonstrate that CamKIIα-positive neurons in the VTA were specifically activated by DREADD.

### Variations of the BOLD signals induced by the activation of CamKIIα-positive neurons in the VTA

**Fig. 4:**
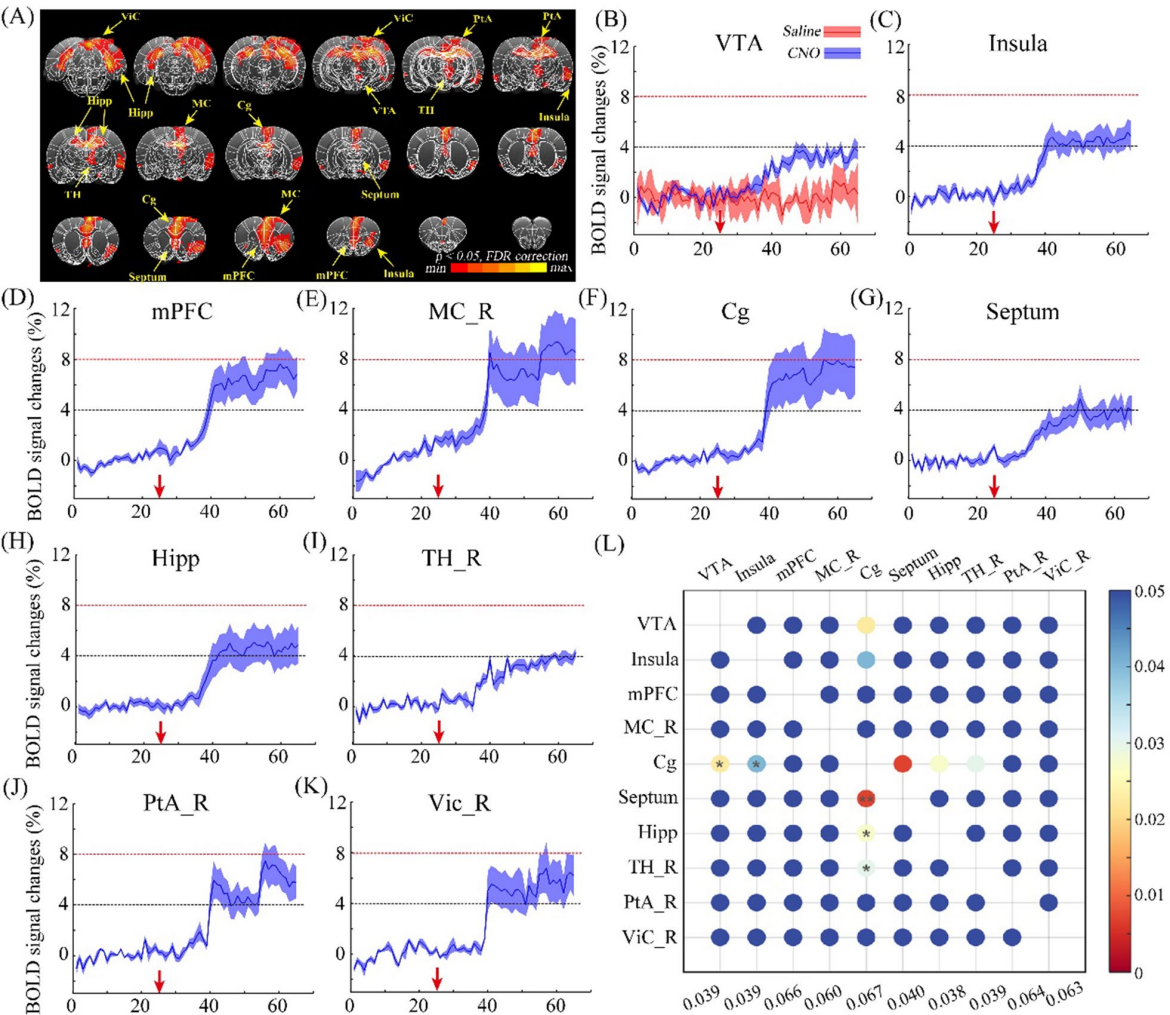
Responses of the BOLD signals following the chemogenetic activation of VTA CamKIIα-positive neurons with the injection of CNO. **A** The Global BOLD signal response, multiple brain regions show significant BOLD signal changes, including the VTA, mPFC, Cg, septum, Hipp, right insula, right TH, right MC, right PtA and right ViC. *N* = *4.* Threshold: FDR, p < 0.05; **B** changes of BOLD signal in the virus infected region (VTA) during chemogenetics activated the CamKII-positive neurons in VTA; red arrow indicates the injection period of CNO (blue line) or saline (red line); **C–K** BOLD signal changes in the regions of Insula (**C**), mPFC (**D**), MC_R (right MC, **E**), Cg (**F**), Septum (**G**), Hipp (**H**), TH_R (right TH, **I**), PtA_R (right PtA, **J**) and Vic_R (right Vic, **K**), red arrow indicates the timepoint of CNO injection; **L** comparison matrix of the BOLD signal increased in the VTA, Insula, mPFC, right MC, Cg, Septum, Hipp, right TH, right PtA and right ViC. The increase values of BOLD signals in the activated brain regions were calculated and compared pairwise. The colors of dots indicate* p*-value. Two-tailed t-test; *p < 0.05; **p < 0.01

The brain-wide neural activity induced by DREADD excitation of VTA CamKIIα-positive neurons was assessed by BOLD-fMRI. The difference in BOLD responses followed by CNO or saline administration is exhibited voxel-by-voxel (Fig. 4A). Changes in neural activity were present in multiple regions, and the time courses of BOLD signals in these brain regions were collected and illustrated in Fig. 4B–K, including the VTA, Insula, mPFC, right motor cortex (MC_R), Cg, Septum, hippocampus (Hipp), right thalamus (TH_R), right parietal association cortex (PtA_R) and right visual cortex (ViC_R). The increase values of BOLD signals in the activated brain regions were quantified and compared using Eq. 1 (Fig. 4L). The results indicated that the BOLD response in the Cg region exhibited a significantly higher magnitude than VTA, Insula, Septum, Hipp and TH_R regions. No significant differences were observed among the BOLD increase values in the remaining regions (Fig. 4L, shown in dark blue). These results imply that the activation of VTA CamKIIα-positive neurons through DREADD stimulation contributed to widespread neural activity in several functional related regions.

### Comparisons of the whole-brain activities caused by stimulation of dopaminergic or CamKIIα -positive neurons in the VTA

**Fig. 5:**
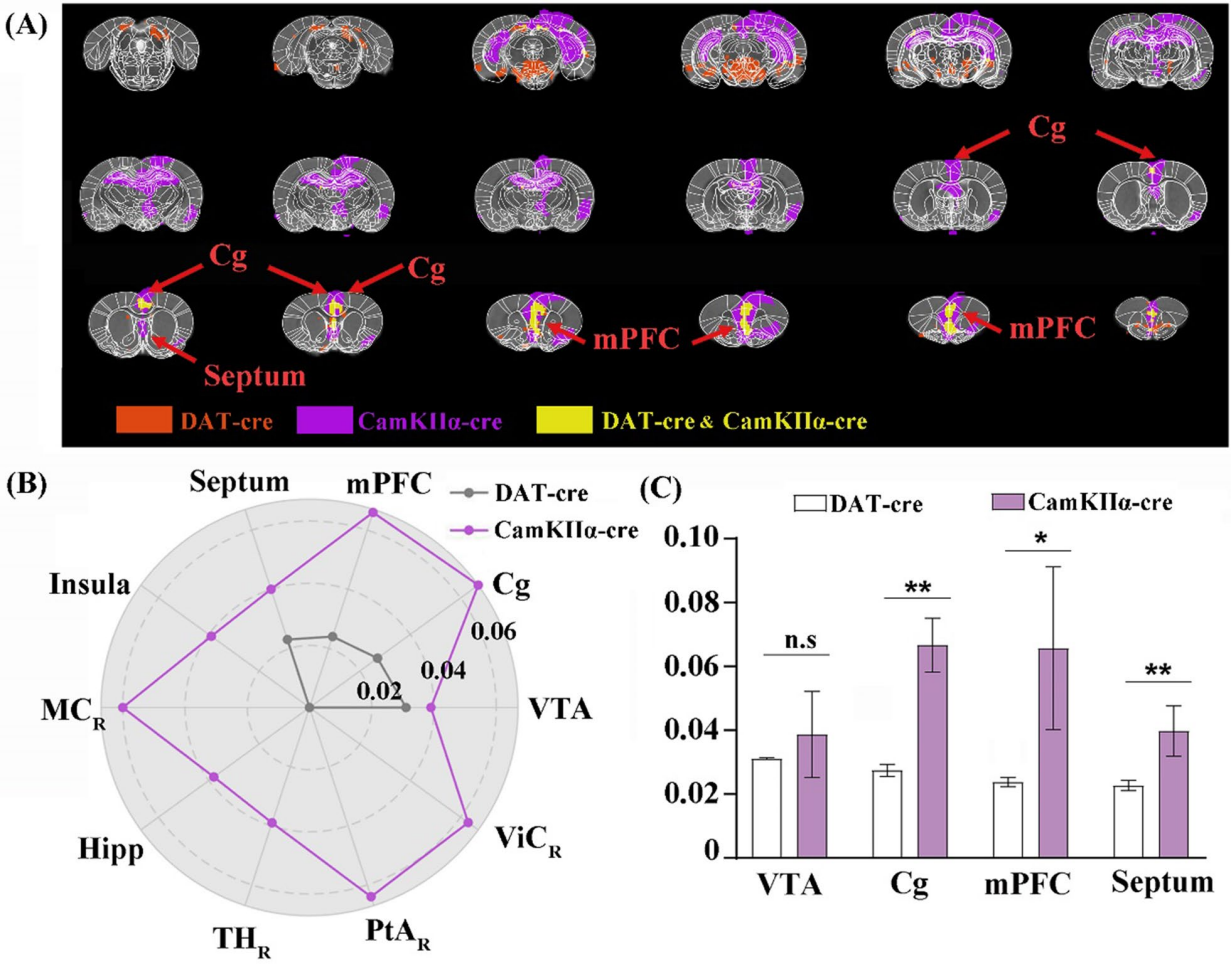
Conjunction analysis of global BOLD signal responses in DAT-cre and CamKIIα-cre rats after the chemogenetic activation in VTA with CNO. **A** Changes of the BOLD signals in both DAT-cre and CamKIIα-cre animals after the chemogenetic activation and the co-localization of the increased BOLD signals were illustrated in yellow, including the Cg, mPFC and Septum; **B** the increased BOLD signals in the cerebral regions after the chemogenetic activation in DAT-cre (gray) or CamKIIα-cre animals (purple); **C** the increased BOLD signals in the co-localizations after the chemogenetic activation in DAT-cre or CamKIIα-cre animals. Two-tailed t-test, Ave. ± STD; *p < 0.05; **p < 0.01

To compare the brain-wide neural activities induced by DREADD stimulation of dopaminergic or CamKIIα-positive neurons in the VTA, the conjunction analysis was performed to identify the brain regions that were activated in both stimulations or in at least one stimulation. The result indicated that Cg, mPFC and Septum were the overlapping brain regions showing BOLD signal changes upon these two different stimulations (Fig. 5A). Even though the VTA exhibited BOLD responses in both stimulations, the overlapping activated voxels were absent in the VTA (Fig. 5A). The increase values of BOLD responses in the VTA and three overlapping brain regions in both stimulations were collected and compared (Fig. 5B, C). The results showed that there was no significant difference between increase values of the VTA in the two stimulations. However, the BOLD responses were stronger in Cg, mPFC and Septum when VTA CamKIIα-positive neurons were DREADD activated.

### Difference between the structural connections of VTA dopaminergic and CamKIIα-positive neurons

**Fig. 6:**
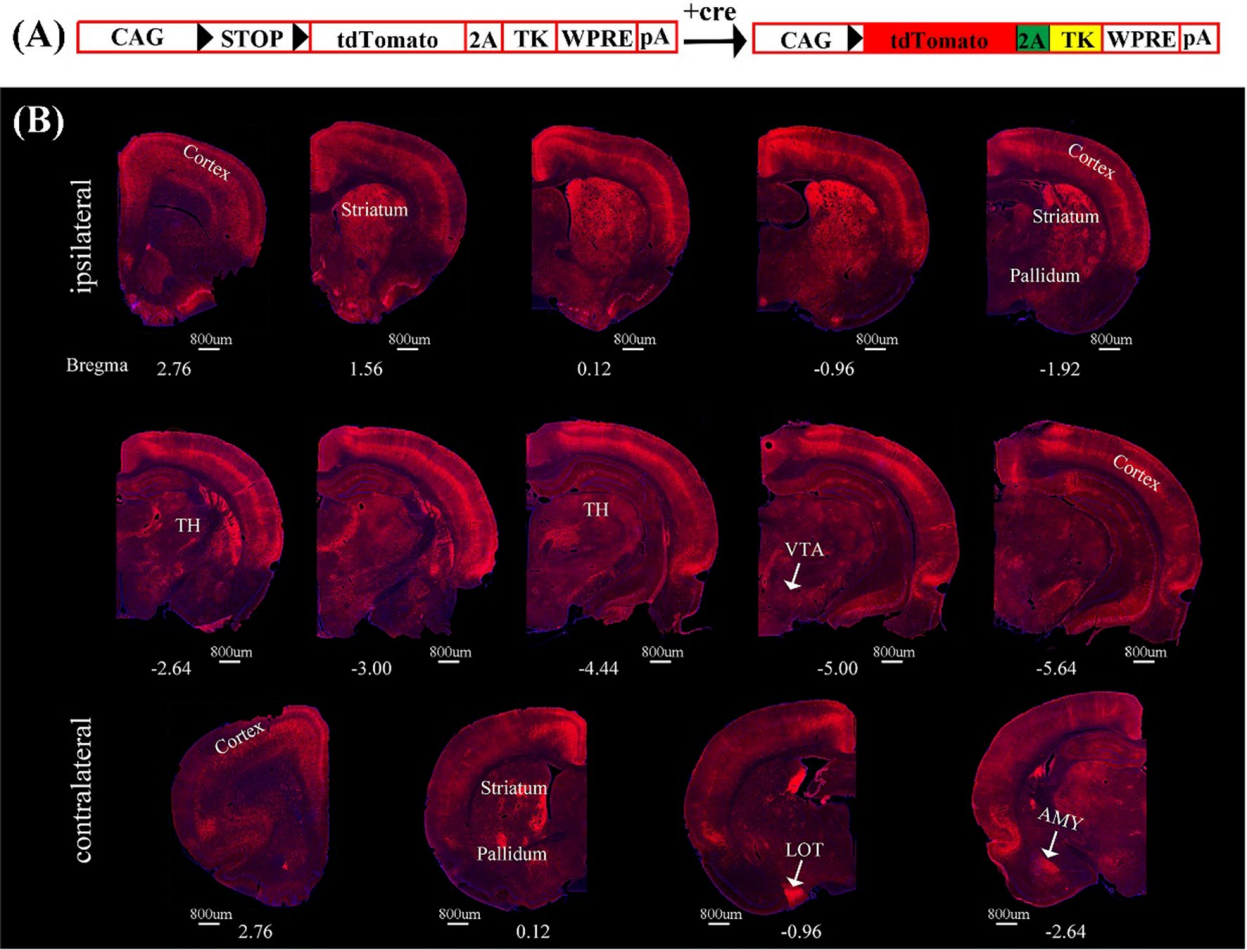
Fluorescence images of representative slices from the whole brain after H129ΔTK-TT injection in the right VTA of DAT-cre rats. **A** The diagram of H129ΔTK-TT virus genome that encoded the Cre-dependent tdTomato (red) and TK gene. Black triangles represent LoxP sites. STOP element prevented the expression of exogenous genes. In the presence of cre, STOP was deleted and tdTomato and TK could be expressed. **B** Cre-dependent td-Tomato expression was observed in the ventral tegmental area (VTA) and its downstream regions, which were labeled in red to emphasize the output network of VTA dopaminergic neurons. The network encompasses the bilateral cerebral cortex, bilateral striatum, bilateral pallidum, ipsilateral TH, ipsilateral Hipp, contralateral LOT and contralateral AMY. The nuclei were stained blue by DAPI. The scale bar: 800 μm

**Fig. 7:**
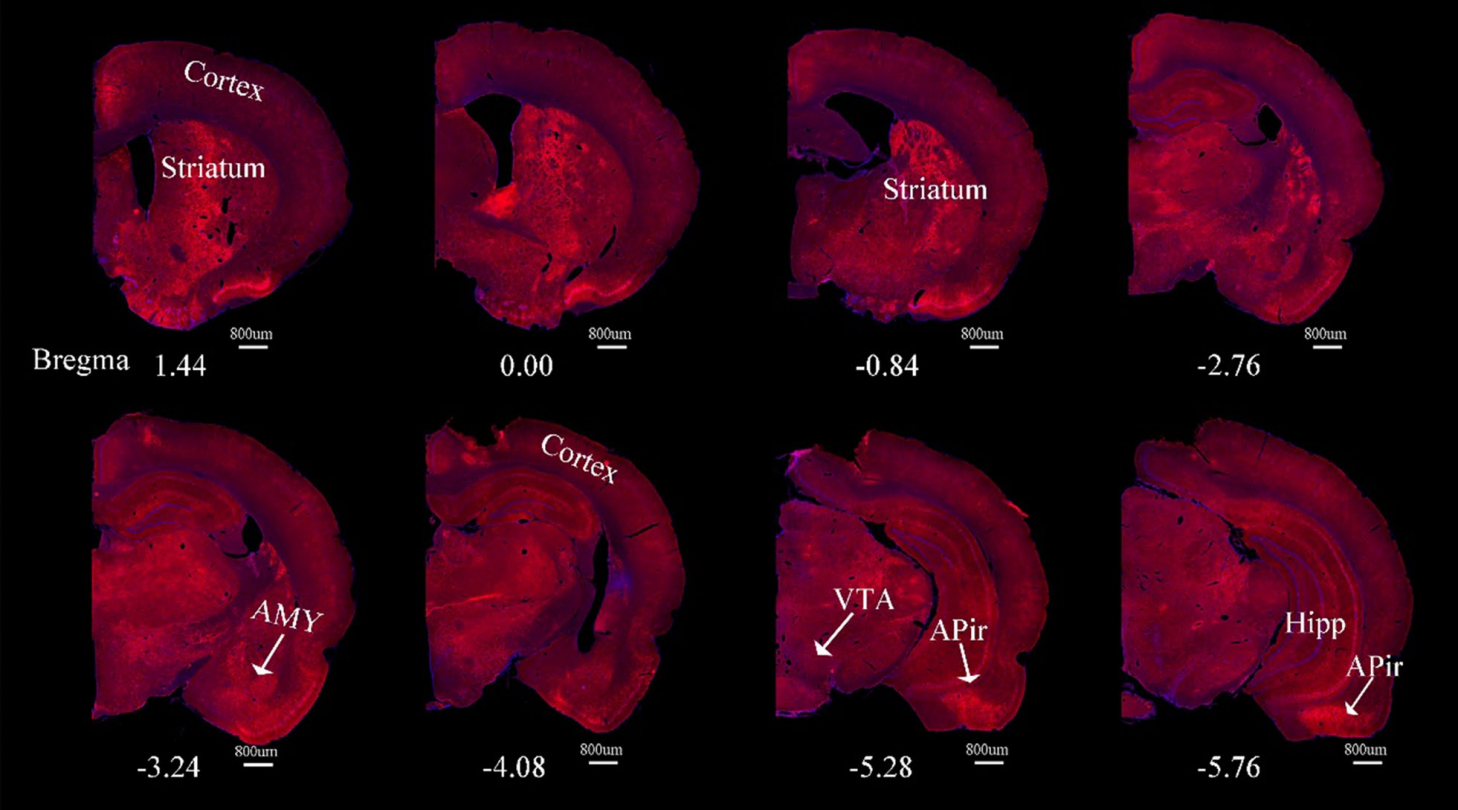
Fluorescence images of representative slices from the whole brain after H129ΔTK-TT injection in the right VTA of CamKIIα-cre rats. The downstream regions of VTA CamKIIα-positive neurons were labeled with tdTomato protein in red, including the cerebral cortex, striatum, AMY, Hipp and Apir. The nuclei were stained blue by DAPI. The scale bar: 800 μm

To investigate the structural downstream networks from VTA dopaminergic or CamKIIα-positive neurons, the anterograde transsynaptic tracer, H129ΔTK-TT was injected into the VTA of DAT-cre or CamKIIα-cre rats (Fig. 6A). The tdTomato reporter, encoded by the viral tracer, was expressed in multiple regions in both groups of rats. The mapping results of DAT-cre rats showed that output networks of VTA dopaminergic neurons included bilateral cerebral cortex, bilateral striatum, bilateral pallidum, ipsilateral TH, ipsilateral Hipp, contralateral nucleus of the lateral olfactory tract (LOT) and contralateral AMY (Fig. 6B). Surprisingly, less regions were labeled in the CamKIIα-cre rats while stimulating VTA CamKIIα-positive neurons elicited large-scale neural activities. The structural downstream regions of VTA CamKIIα-positive neurons were abundance in the ipsilateral sphere, including cerebral cortex, striatum, AMY, Hipp and amygdalopiriform transition area (Apir) (Fig. 7).

## Discussion

DREADD-fMRI could causally link the chemogenetic activation of local genetically targeted neurons with changes in brain-wide neural networks. In the current study, DREADD-fMRI was utilized to assess the functional neural networks of dopaminergic and CamKIIα-positive neurons in the region of VTA. To investigate the structural neural networks of these two neuron subpopulations, the trans-multi-synaptic HSV was applied to detect the downstream networks of dopaminergic and CamKIIα-positive neurons in the VTA. Results of structural and functional neural networks of different cell-type subpopulations in the VTA were analyzed and compared.

### Relationships of BOLD signal variations and activation of the dopaminergic neurons

Our study demonstrated that the chemogenetic activation of VTA dopaminergic neurons elicited distinct BOLD responses only in the VTA and a few relevant brain regions. Some brain regions receiving VTA dopaminergic neuron projections did not exhibit BOLD signaling changes such as NAcc and olfactory tubercle. Previous research has shown that dopamine release but not BOLD activity was observed in NAcc when the dopaminergic neurons in the VTA were stimulated by optogenetics [20]. This finding is consistent with our results despite the different stimulation modality. This phenomenon might be related with the differential effects of dopamine on the postsynaptic neurons. Dopamine is an important neurotransmitter and there are two main classes of dopamine receptors, D1 and D2. The D1 receptors are Gs/a coupled and can stimulate neurons by activating cyclic AMP-dependent protein kinase, whereas the D2 receptors are Gi coupled and have an opposite effect [36]. Both D1 and D2 receptor-expressing neurons in NAcc are heavily innervated by dopaminergic neurons from the VTA. The dopamine release in NAcc can simultaneously cause the activation of D1 receptor positive neurons and inhibition of D2 receptor positive neurons. This could be the reason why the BOLD response that reflects the average activity across local neural populations was not detected in NAcc or other regions innervated by VTA dopaminergic neurons. According to previous reports and our present study, the release of dopamine does not reliably predict the BOLD signal change, and the distribution of various dopaminergic receptors in the investigated regions should be taken into consideration.

### Relationships of BOLD signals and activation of CamKIIα positive neurons

There have been reports indicating that optogenetic activation of VTA CamKIIα-positive neurons (mainly glutamatergic and some dopaminergic neurons) can elicit a wide range of increases in BOLD signals throughout the brain [20, 25]. This is basically consistent with our results despite the different activation modality. There is an overlap between the stimulation of VTA CamKIIα-positive and dopaminergic neurons. A subset of CamKIIα-positive neurons is dopaminergic and the dopaminergic neurons that are CamKIIα-positive could also be stimulated in the DAT-cre animals. According to previous studies [19, 20, 25], stimulation targeting CamKIIα-positive VTA neurons, comprising both glutamatergic and dopaminergic cells, is ‘less-specific’, and the stimulation of dopaminergic neurons in DAT-cre rats is more specific. Their results also suggest that blockade of dopaminergic D1/5 receptors by SCH23390 does not attenuate BOLD responses evoked by optogenetic activation of VTA CamKIIα-positive neurons. Thus, we speculated that the BOLD responses in this situation were mainly driven by glutamate. They also reported that application of glutamatergic NMDA receptor blocker (MK801) leads to a distinct decrease in BOLD signals caused by electrical stimulation of the VTA, which also reveals the important roles of glutamatergic neurons and glutamatergic receptors in driving BOLD responses.

### Comparisons of the BOLD signals induced by the stimulation of dopaminergic or CamKIIα-positive neurons

The brain-wide neural activities induced by DREADD stimulation of dopaminergic or CamKIIα-positive neurons in the VTA were compared. The results suggest that Cg, mPFC and Septum are the overlapping brain regions that showed BOLD signal changes upon these two different stimulations. Moreover, BOLD responses were stronger in Cg, mPFC and Septum when VTA CamKIIα-positive neurons were DREADD activated. The differential extent and intensity of BOLD responses to the two stimulations could be associated with the number and type of activated neurons in the functional-related regions. The composition of glutamatergic and dopaminergic output from VTA can result in differential or overlapping responsive brain regions between two cre strains. For example, previous studies revealed that VTA glutamatergic and dopaminergic neurons differentially innervate PFC. The proportionality of VTA glutamatergic neurons projecting to the PFC is higher than dopaminergic neurons [37]. This could be a reason why CamKIIα-positive neurons stimulation induced stronger and broader BOLD responses in mPFC.

### Relationships of the structural and functional networks of the specific cell-type neurons in the VTA

The genetically-engineered neurotropic viral vectors have been widely used in neural circuit tracing and significantly advance our knowledge of neural connections among different brain regions. Using virus-based tracing tools, the previous studies have demonstrated that VTA dopaminergic neurons directly project to hippocampus, prefrontal cortex (PFC), NAcc and amygdala [3, 38, 39] and VTA glutamatergic neurons project to hippocampus, NAcc, lateral hypothalamic area (LH) and VP [17, 40, 41]. HSV derived vectors are effective tools for mapping the neural circuit of rodents and non-human primates since they have high capacity and wide host range [42–44]. The herpes simplex virus type 1 (HSV-1) 129 (H129) strain has been demonstrated to be an anterograde multi-synaptic neuronal circuit tracer and has been employed to map the output neural networks in different animal models [45, 46]. For instance, the EGFP-encoding H129 vector (H129-EGFP) was utilized to infect the upper respiratory tract of animals and trace the relevant sensory pathway [47–49]. To trace the synaptic outputs from specific type neurons, a cre recombinase-dependent H129 derived tracer, H129-ΔTK-TT was developed. The endogenous H129 viral Thymidine Kinase (TK) gene was replaced with a cre-dependent TK expressing cassette, so as to enable the tracing of the output neural circuit of genetically targeted neurons expressing cre [31]. In the current study, the trans-multi-synaptic outputs of VTA dopaminergic or CamKIIα-positive neurons which include mainly glutamatergic and some dopaminergic neurons, were elucidated by H129ΔTK-TT. The output structural networks of VTA dopaminergic neurons cover multiple different cortical and subcortical brain regions including the functional-related regions revealed by DREADD-fMRI. These results verified the structural basis for the functional connectivity. However, not all brain regions exhibiting BOLD responses upon stimulation of VTA CamKIIα-positive neurons were labeled by the H129ΔTK-TT. For instance, DREADD stimulating VTA CamKIIα-positive neurons drove BOLD signal changes over a large area of the dorsal Hipp while few neurons in the dorsal Hipp showed fluorescent signal caused by H129ΔTK-TT infection. It is possible that of HSV has different tropisms to infect different types of neurons [50, 51]. Some brain regions that are connected to VTA CamKIIα-positive neurons may be less permissive for HSV-1 infection thereby the fluorescent signals were relatively weak or invisible in these regions.

## Limitations and perspective

Here, VTA CamKIIα-positive and dopaminergic neurons were targeted and stimulated using DREADD-fMRI and neurotropic virus tracing techniques. The functional and structural networks of VTA were explored and compared, respectively. It is worth noting that employing different transgenic animals and genetically manipulated neurotropic viruses could potentially reveal additional neural network findings in various brain regions and specific neuron subpopulations. However, it is important to acknowledge that the two types of networks were analyzed separately in the states of either in vivo or in vitro states, which limits direct comparisons between these two networks. To overcome this limitation, the development of in vivo technologies capable of mapping both functional and structural networks is crucial. With the discovery and application of various MRI reporter genes [52–54], the in vivo detection of the expressing of the neurotropic virus in the brain has been achieved, even for different kinds of neural cells [55–57]. This advancement opens up the possibility of simultaneously detecting functional and structural networks in vivo, which is crucial for understanding the underlying structural basis of functional networks. Moreover, by operating specific neural cells in vivo and recording changes in BOLD signals, it is feasible to evaluate the contributions of astrocytes, neurons, and vasculature for the BOLD signal. This integrated approach has the potential to provide valuable insights into the interpretation of BOLD signals in the brain and enhance our understanding of the intricate interplay between different cellular components during neural activity.

## Conclusions

In the current study, DREADD-fMRI was employed to investigate the functional neural networks of dopaminergic or CamKIIα-positive neurons in VTA region. DREADD activation of VTA dopaminergic neurons induced neural activity in the VTA itself and off-target functional related regions including mPFC, Cg and Septum. Likewise, DREADD stimulation of VTA CamKIIα-positive neurons elicited changes of neural activity in multiple regions including the VTA, Insula, mPFC, MC_R, Cg, Septum, Hipp, TH_R, PtA_R and ViC_R. In addition, the trans-multi-synaptic HSV was applied to detect the downstream structural neural networks of dopaminergic or CamKIIα-positive neurons in the VTA. Expectedly, the virus-labeled output structural networks of VTA dopaminergic neurons cover multiple cortical and subcortical brain regions including the functional-related regions revealed by DREADD-fMRI. However, some brain regions that exhibit BOLD responses upon stimulation of VTA CamKIIα-positive neurons were not labeled by the viral tracer. The comparative dissection of structural and functional neural networks of different cell-type subpopulations in the VTA will deepen our understanding of the brain-wide VTA related neural networks.

### Supplementary Information

**Additional file 1: Methods.** Animal behavioral testing. **Figure S1.** Expression of hM3D(Gq) in CamKIIα-positive neurons in the VTA. (**A**) the cre-dependent neurons expressing hM3D (Gq) and mCherry proteins (red); (**B**) CamKIIα neurons were stained by the antibody of Anti-CamKII (green); (**C**) co-locations of the CamKIIα neurons (yellow) by merging the results of the virus labeling (**A**) and immunohistochemistry (**B**). Panels (**D**), (**E**), (**F**) are higher-magnification images of boxed regions in (**A**), (**B**), (**C**), respectively. The nuclei were stained blue by DAPI, and the scale bar: 200 μm. **Figure S2.** Immunofluorescent c-Fos staining in the VTA of CamKIIα-cre rats infected with hM3D(Gq) encoding rAAV after the activation with CNO (chemogenetic activation) or saline. (**A**–**C**) Representative coronal sections show the virus-labeled CamKIIα neurons (red, **A**), and the immunofluorescent CamKIIα neurons with c-Fos staining (green, **B**) and the co-localization neurons (yellow, **C**) of in the VTA after the chemogenetics activation of VTA CamKIIα neurons; Panels **A1**, **B1** and **C1** are higher-magnification images of the boxed regions in panels of **A**, **B** and **C**, respectively; (**D**–**F**) the saline injection did not cause obvious c-Fos signal in the region of VTA of CamKIIα-cre transgenic rats; (**D**) virus-labeled CamKIIα neurons (red); (**E**) results of immunofluorescent c-Fos staining; (**F**) the merged results of the virus labeling (**D**) and c-Fos immunohistochemistry (**E**); Panels **D1**, **E1** and **F1** are higher-magnification images of boxed regions in the panels of **D**, **E** and **F**, respectively. Note: The nuclei were stained blue by DAPI. The scale bar: 200 μm. **Figure S3**. Chemogenetics activation of dopaminergic neurons in VTA resulted in hyperactivity. (**A**, **D**) The heatmap illustrates the position of the animals in the open field after the injection of CNO (**A**) or Saline (**D**). The color gradient from blue to red represents the duration of time the animal spends in a particular position, with red indicating a longer duration. (**B**–**C**) The distance traveled after CNO injection was significantly higher compared to the distance traveled after saline injection (**B**), there was no significant difference in the duration traveled (**C**). (**E**–**F**) There was no significant difference in locomotion distance (**E**) or duration (**F**) before the injection of CNO and Saline. *N* = 5, Two-tailed t-test, Ave. ± STD; *p < 0.05; **p < 0.01. **Figure S4**. Chemogenetics activation of CamKIIα-positive neurons in the VTA resulted in hyperactivity. (**A**, **D**) The heatmap depicts the animal’s position in the open field after the injection of CNO (**A**) or Saline (**D**). (**B**–**C**) The distance (**B**) and duration (**C**) traveled after CNO injection were significantly higher compared to those after saline injection. (**E**–**F**) There were no significant differences in locomotion distance (**E**) or duration (**F**) before the injection of CNO and Saline. Note: *N* = 5, Two-tailed t-test, Ave. ± STD; *p < 0.05; **p < 0.01.

## Data Availability

The datasets in the current study are available from the corresponding author on reasonable request.
