# Supplementary material for: Investigations of brain-wide functional and structural networks of dopaminergic and CamKIIα-positive neurons in VTA with DREADD-fMRI and neurotropic virus tracing technologies
Source: J Transl Med. 2023 Aug 14;21:543. doi: 10.1186/s12967-023-04362-6 (PMC10424380; doi:10.1186/s12967-023-04362-6)
Supplement: Supplementary file 1 — Additional file 1: Methods. Animal behavioral testing. Figure S1. Expression of hM3D(Gq) in CamKIIα-positive neurons in the VTA. (A) the cre-dependent neurons expressing hM3D (Gq) and mCherry proteins (red); (B) CamKIIα neurons were stained by the antibody of Anti-CamKII (green); (C) co-locations of the CamKIIα neurons (yellow) by merging the results of the virus labeling (A) and immunohistochemistry (B). Panels (D), (E), (F) are higher-magnification images of boxed regions in (A), (B), (C), respectively. The nuclei were stained blue by DAPI, and the scale bar: 200 μm. Figure S2. Immunofluorescent c-Fos staining in the VTA of CamKIIα-cre rats infected with hM3D(Gq) encoding rAAV after the activation with CNO (chemogenetic activation) or saline. (A–C) Representative coronal sections show the virus-labeled CamKIIα neurons (red, A), and the immunofluorescent CamKIIα neurons with c-Fos staining (green, B) and the co-localization neurons (yellow, C) of in the VTA after the chemogenetics activation of VTA CamKIIα neurons; Panels A1, B1 and C1 are higher-magnification images of the boxed regions in panels of A, B and C, respectively; (D–F) the saline injection did not cause obvious c-Fos signal in the region of VTA of CamKIIα-cre transgenic rats; (D) virus-labeled CamKIIα neurons (red); (E) results of immunofluorescent c-Fos staining; (F) the merged results of the virus labeling (D) and c-Fos immunohistochemistry (E); Panels D1, E1 and F1 are higher-magnification images of boxed regions in the panels of D, E and F, respectively. Note: The nuclei were stained blue by DAPI. The scale bar: 200 μm. Figure S3. Chemogenetics activation of dopaminergic neurons in VTA resulted in hyperactivity. (A, D) The heatmap illustrates the position of the animals in the open field after the injection of CNO (A) or Saline (D). The color gradient from blue to red represents the duration of time the animal spends in a particular position, with red indicating a longer duration. (B–C) The distanc [file 12967_2023_4362_MOESM1_ESM.docx]

**Supplementary Materials**

**Animal behavioral testing**

The open field behavioral testing was recorded in a box (100×100×40 cm^3^) with black inner wall and floor. An infrared digital camera was mounted directly above it with a field of view covering the entire open field, and the behavioral data was recorded and analyzed using the Anymaze software (Stoelting, USA). On the experimental day, the animal was placed in the center of the open field box and the Anymaze recording system was turned on simultaneously. The free movement of the rat was recorded for 15 minutes. Then, the rat was intraperitoneally injected with saline or 1 mg/ml CNO (2 mg/kg), and the rat was immediately placed back to the center of the open field box again. The Anymaze recording system was turned on and recorded the free movement for one hour after the injection. After the recording of each animal, the inner wall and bottom of the behavior box were cleaned to prevent the smell left by the previous animal from affecting the moving of the later animal. The behavior experiments were performed in a quiet room with appropriate temperature and ventilation, and all the animals were injected with CNO or saline at the same time point on different days.

**Table S1. The antibody information used in Immunohistochemistry.**

| Antibody Name | Company | Art.No | Dilution Ratio |
| --- | --- | --- | --- |
| Anti-TH | abcam | 2552365 | 1:1000 |
| Anti-CamKII | abcam | ab5683 | 1:500 |
| Anti-cfos | cell signaling | 2250 | 1:500 |
| Anti-dsred | takara | 632496 | 1:1000 |
| Goat Anti-Rabbit-488 | Jackson | 111302 | 1:200 |
| Goat Anti-Rabbit-cy3 | Jackson | 111-165-003 | 1:400 |


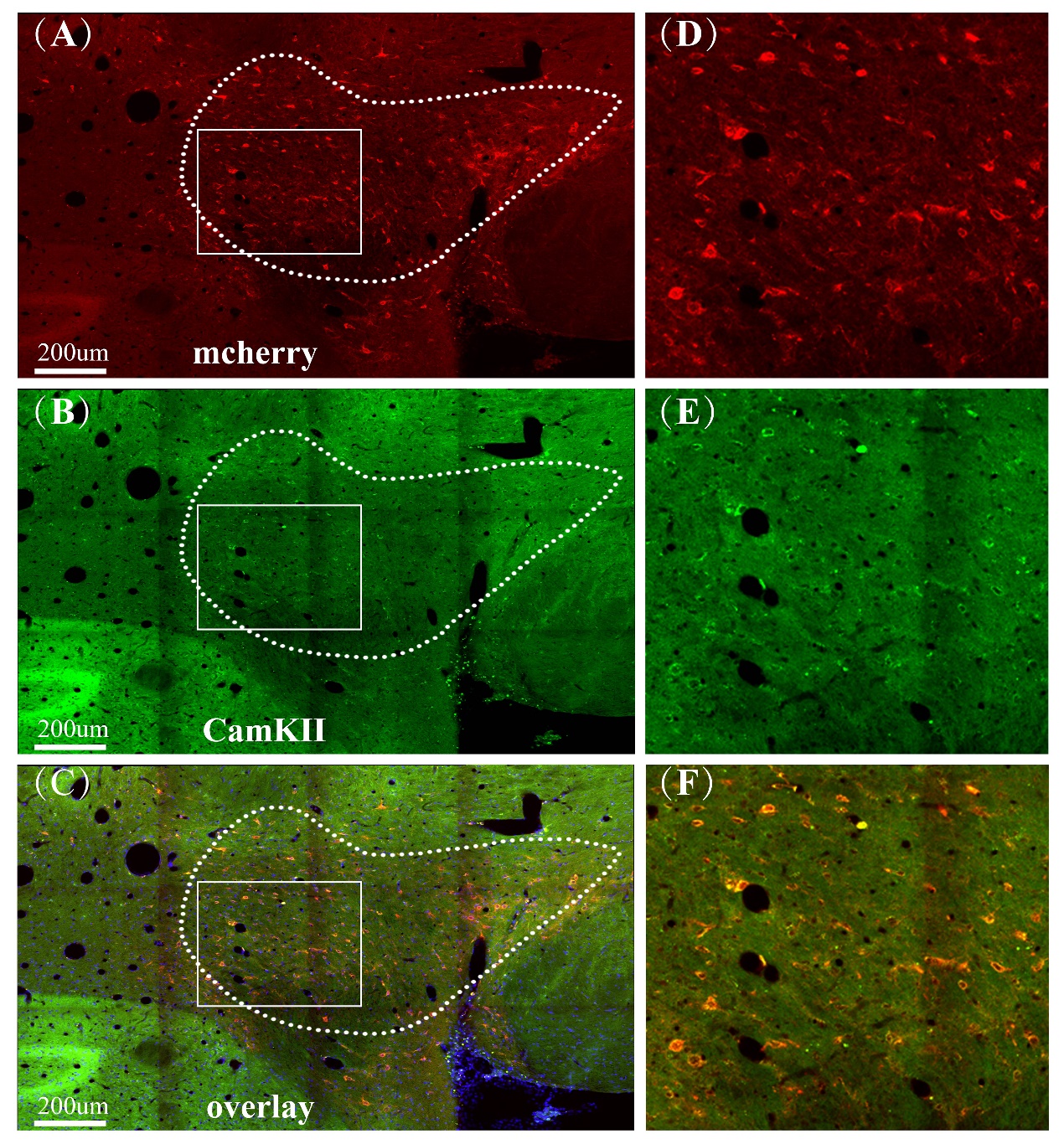


**Figure S1. Expression of hM3D(Gq) in CamKIIα-positive neurons in the VTA. (A)** the cre-dependent neurons expressing hM3D (Gq) and mCherry proteins (red); **(B)** CamKIIα neurons were stained by the antibody of Anti-CamKII (green); **(C)** co-locations of the CamKIIα neurons (yellow) by merging the results of the virus labeling **(A)** and immunohistochemistry **(B)**. Panels **(D)**, **(E)**, **(F)** are higher-magnification images of boxed regions in **(A)**, **(B)**, **(C)**, respectively. The nuclei were stained blue by DAPI, and the scale bar: 200 μm.


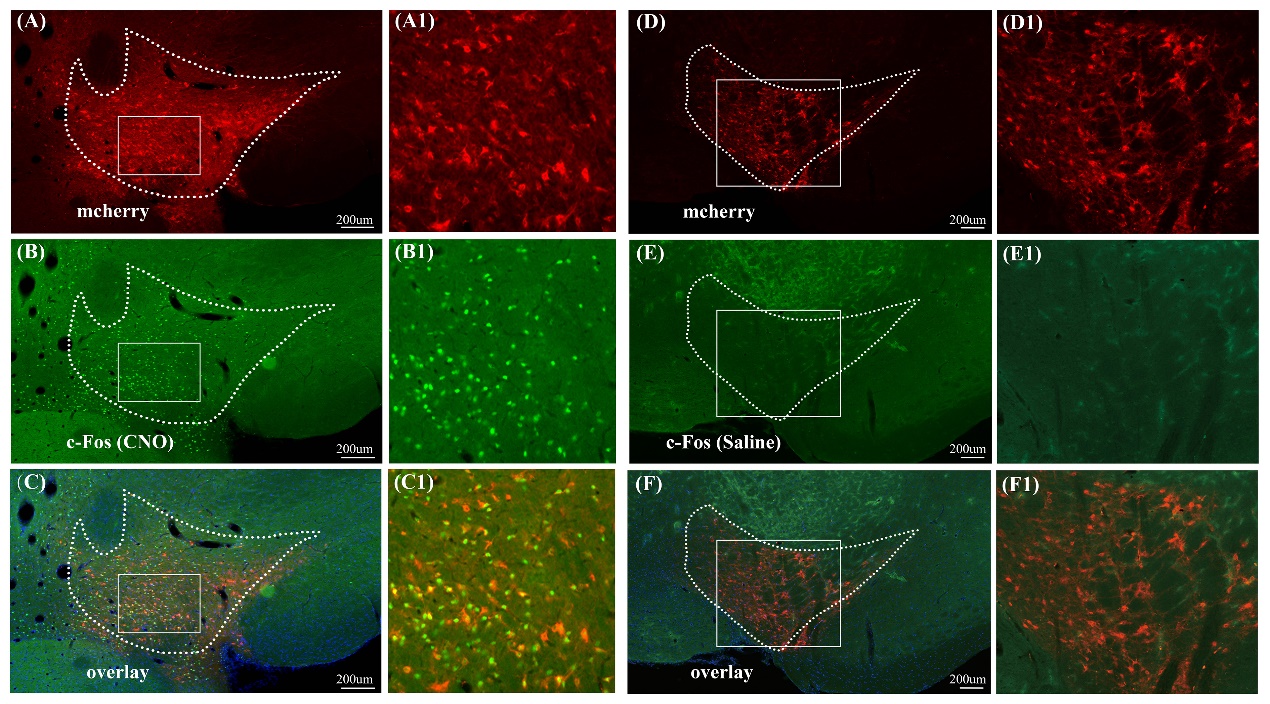


**Figure S2. Immunofluorescent c-Fos staining in the VTA of CamKIIα-cre rats infected with hM3D(Gq) encoding rAAV after the activation with CNO (chemogenetic activation) or saline. (A-C)** Representative coronal sections show the virus-labeled CamKIIα neurons (red, **A**), and the immunofluorescent CamKIIα neurons with c-Fos staining (green, **B**) and the co-localization neurons (yellow, **C**) of in the VTA after the chemogenetics activation of VTA CamKIIα neurons; Panels **A1**, **B1** and **C1** are higher-magnification images of the boxed regions in panels of **A**, **B** and **C**, respectively; **(D-F)** the saline injection did not cause obvious c-Fos signal in the region of VTA of CamKIIα-cre transgenic rats; **(D)** virus-labeled CamKIIα neurons (red); **(E)** results of immunofluorescent c-Fos staining; **(F)** the merged results of the virus labeling **(D)** and c-Fos immunohistochemistry **(E)**; Panels **D1**, **E1** and **F1** are higher-magnification images of boxed regions in the panels of **D**, **E** and **F**, respectively. *Note: The nuclei were stained blue by DAPI. The scale bar: 200 μm.*


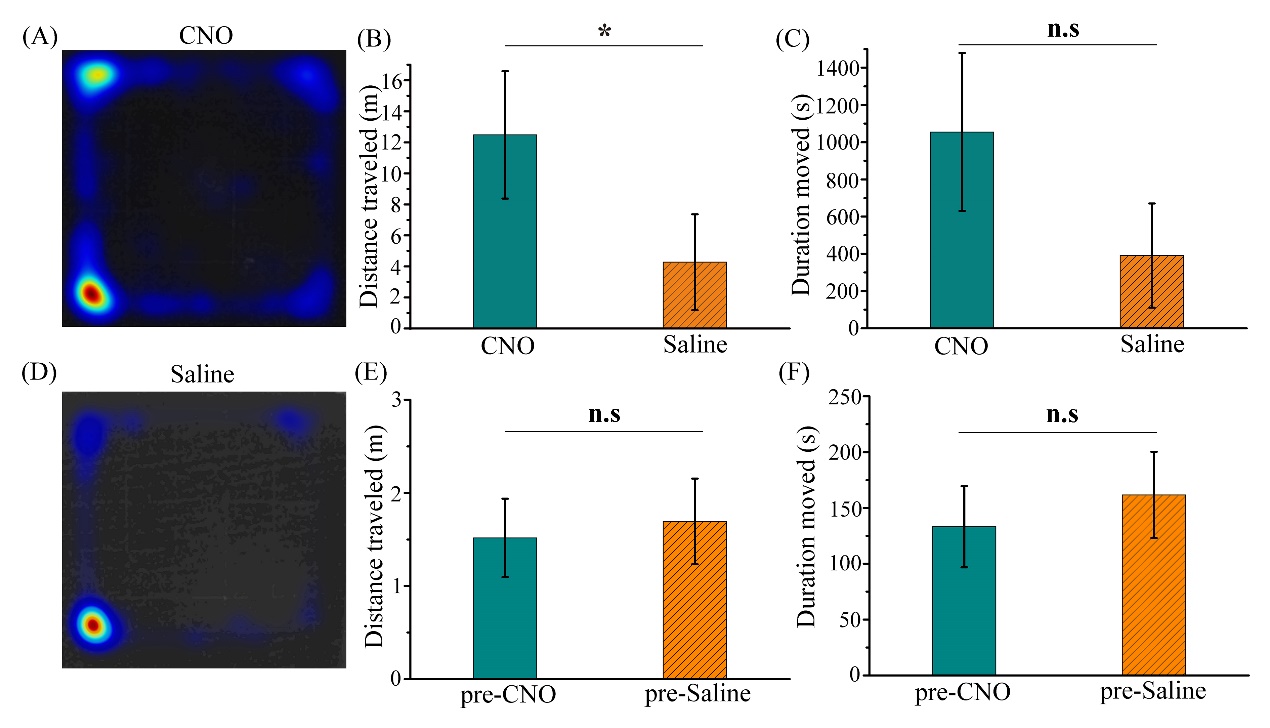


**Figure S3. Chemogenetics activation of dopaminergic neurons in VTA resulted in hyperactivity.** **(A, D)** The heatmap illustrates the position of the animals in the open field after the injection of CNO **(A)** or Saline **(D)**. The color gradient from blue to red represents the duration of time the animal spends in a particular position, with red indicating a longer duration. **(B-C)** The distance traveled after CNO injection was significantly higher compared to the distance traveled after saline injection **(B)**, there was no significant difference in the duration traveled **(C)**. **(E-F)** There was no significant difference in locomotion distance **(E)** or duration **(F)** before the injection of CNO and Saline. N = 5, Two-tailed t-test, Ave. ± STD; *: p<0.05.


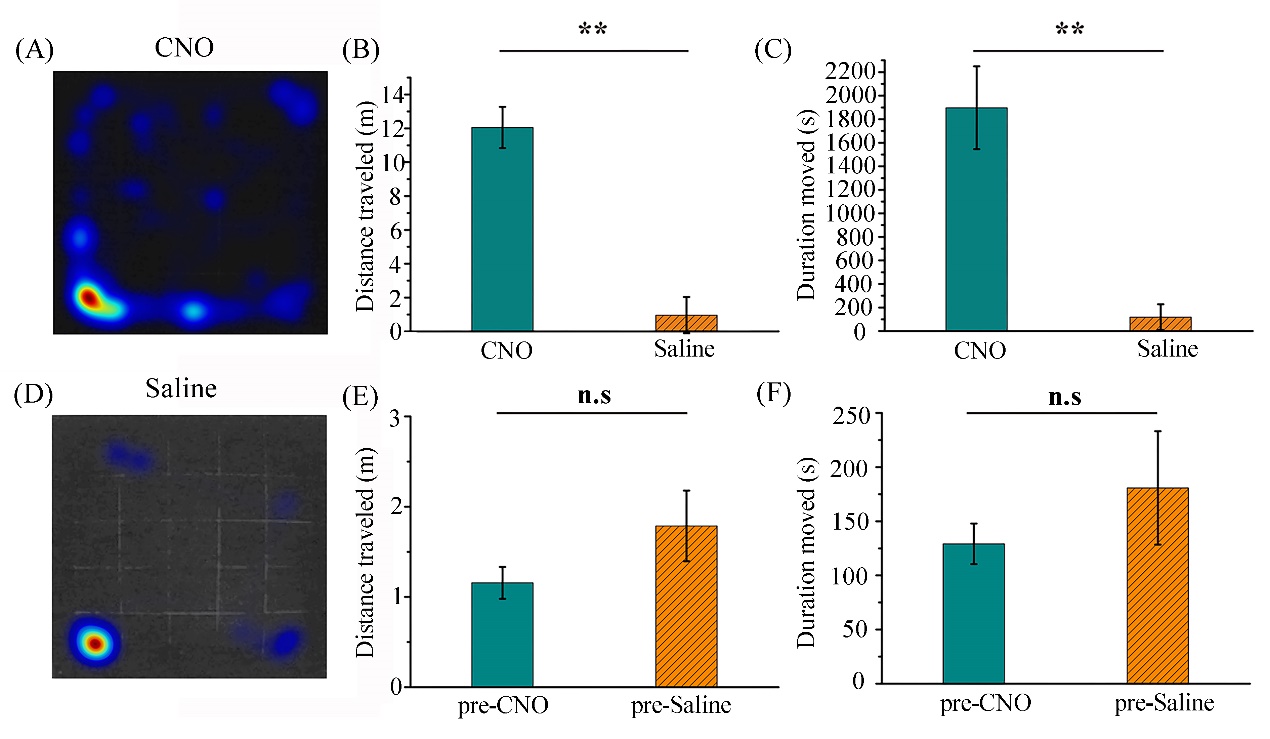


**Figure S4. Chemogenetics activation of CamKIIα-positive neurons in the VTA resulted in hyperactivity. (A, D)** The heatmap depicts the animal's position in the open field after the injection of CNO **(A)** or Saline **(D)**. **(B-C)** The distance **(B)** and duration **(C)** traveled after CNO injection were significantly higher compared to those after saline injection. **(E-F)** There were no significant differences in locomotion distance **(E)** or duration **(F)** before the injection of CNO and Saline. *Note: N = 5, Two-tailed t-test, Ave. ± STD; *: p<0.05; **p<0.01.*
